# Tracheal Papilloma Treated with Cryotherapy and Interferon-*α*: A Case Report and Review of the Literature

**DOI:** 10.1155/2015/356796

**Published:** 2015-02-18

**Authors:** Fatma Yıldırım, Murat Türk, Sedat Demircan, Nalan Akyürek, Ahmet Selim Yurdakul

**Affiliations:** ^1^Department of Pulmonary Medicine, Gazi University Faculty of Medicine, Ankara, Turkey; ^2^Department of Thoracic Surgery, Gazi University Faculty of Medicine, Ankara, Turkey; ^3^Department of Pathology, Gazi University Faculty of Medicine, Ankara, Turkey

## Abstract

Tracheal papilloma (TP) is characterized by papillomatous growth of the bronchial epithelium that involves the trachea as a response to Human Papilloma Virus (HPV) infection. A 40-year-old male, with 3-month history of progressive dyspnea was admitted to our hospital, and there were no any other respiratory symptoms. Physical examination was unremarkable. Chest computed tomography (CT) showed that there was a papillomatous mass at the distal trachea. The lesion occupied 80% of tracheal lumen. This patient received cryotherapy and mechanical debridement under general anesthesia and postoperative pathology showed endotracheal papillomatosis. Patient was treated with interferon-*α* (IFN-*α*) and he showed no recurrence at the 8th month of his therapy.

## 1. Introduction

Tracheal papilloma (TP) is a neoplastic lesion secondary to tracheal manifestation of recurrent respiratory papillomatosis (RRP) and characterized by papillomatous growth of the bronchial epithelium as a response to Human Papilloma Virus (HPV) infection. Recurrent bronchoscopic interventions and adjuvant medical therapy are needed in its management. It should be closely followed up since malign transformation is reported especially in smokers. Here we present a case of solitary tracheal papilloma that underwent bronchoscopic endobronchial therapy and had no recurrence during follow-up under medical therapy.

## 2. Case

**Figure 1:**
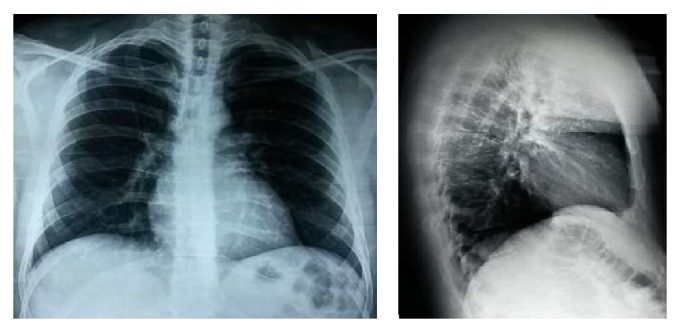
Posteroanterior and lateral chest X-rays of the patient.

**Figure 2:**
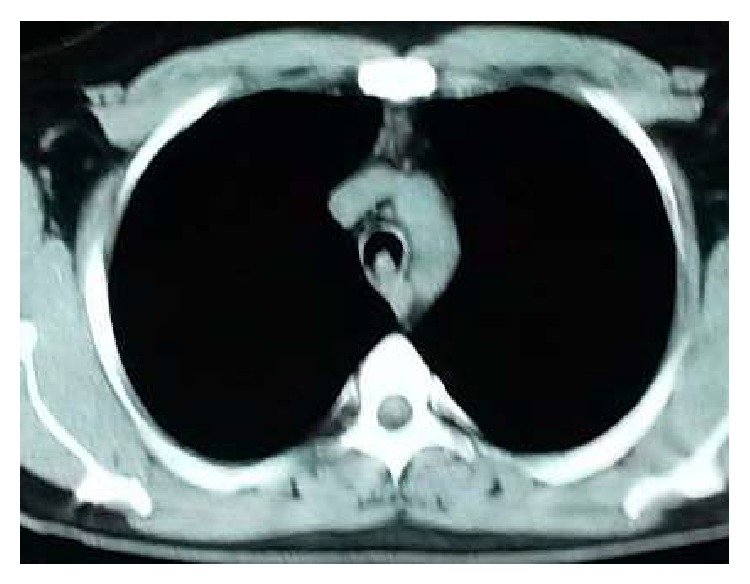
Chest CT showed a lesion protruding to the lumen in distal trachea at the posterior wall.

**Figure 3:**
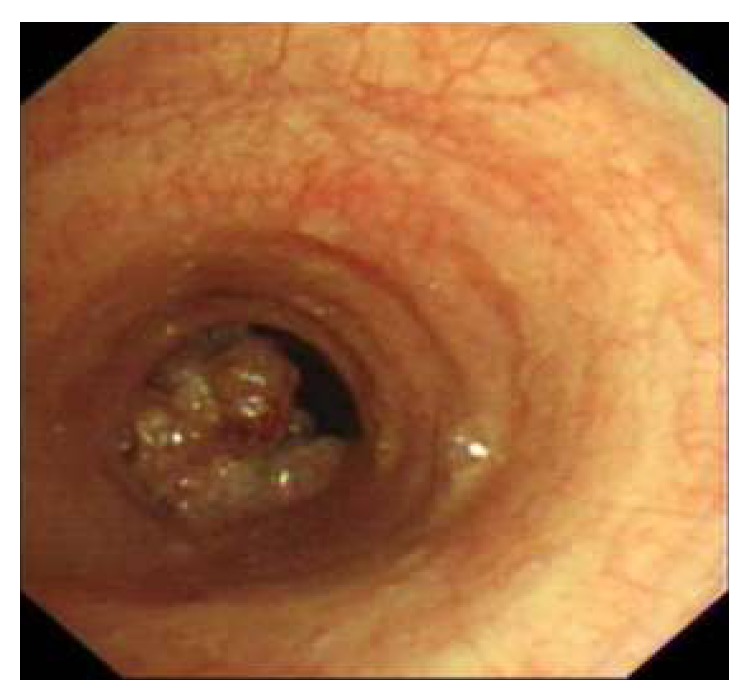
Bronchoscopic view of the lesion, causing 80% obstruction at the tracheal lumen.

40-year-old, active smoker male presented with progressive shortness of breath for the last three months. He had no other respiratory or systemic symptoms. His physical examination was unremarkable other than decreased lung sounds. Routine blood tests and chest X-ray were normal (Figure 1). At chest computed tomography (CT) a mass was seen at the distal trachea, nearly obstructing the lumen (Figure 2). At fiberoptic bronchoscopic (FOB) evaluation, there was a papillomatous lesion at the posterolateral wall of the distal 1/3 of trachea, causing ~80% obstruction at the lumen (Figure 3). The lesions were extracted under general anesthesia via endobronchial therapy by rigid bronchoscopy (mechanical debridement and cryotherapy).

**Figure 4:**
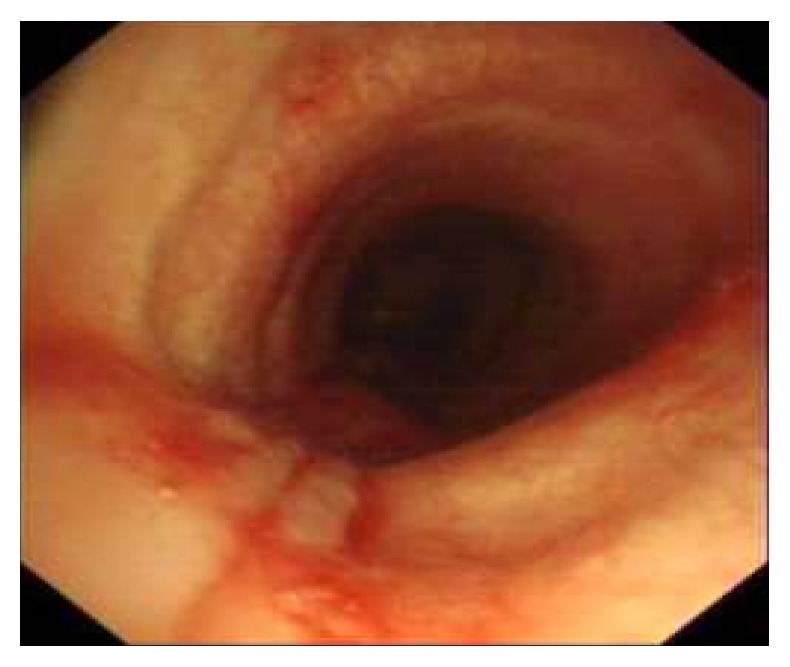
Bronchoscopic view, tracheal wall after excision of the lesion with cryotherapy.

**Figure 5:**
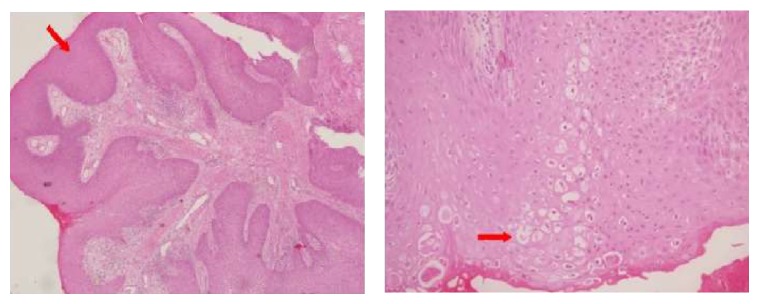
Papillary structures made by hyperplastic squamous epithelium are seen in fibrovascular core (H&E, ×40), and koilocytic cells with perinuclear vacuolisation are seen in hyperplastic squamous epithelium (H&E, ×200).

In the pathology, squamous epithelial hyperplasia of bronchial epithelium with small papillomatous structures was seen (Figure 4). There was perinuclear cytoplasmic vacuolisation (koilocytosis) in some cells at the superficial aspect of the epithelium. There were no dysplastic changes in the epithelial cells (Figure 5). Immunohistochemical studies for determining etiology were positive for HPV. Polymerase chain reaction amplification detected HPV type 6 DNA in the papilloma tissue.

**Figure 6:**
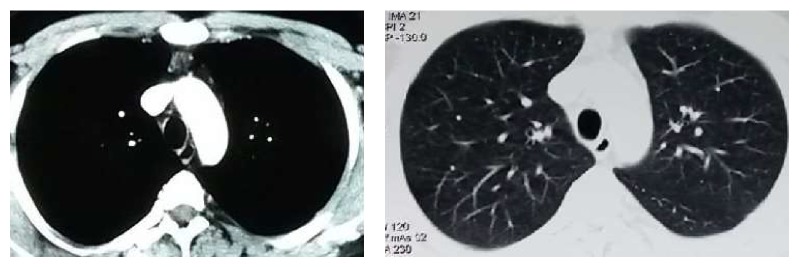
CT sections at the 6th month: tracheal lumen is normal.

Subcutaneous interferon-2*α* (IFN-*α*) treatment was started in doses of 3 million units per square meter 3 times per week. Computed tomography and bronchoscopic evaluation at the 3rd month of the treatment showed no recurrence (Figure 6) and IFN-*α* treatment was continued for 6 months. The patient is still under close follow-up at the 8th month of his diagnosis without recurrence.

## 3. Discussion

Tracheal papilloma (TP) is a rare tumor of trachea consisting of 0.38% of all lung tumors. It can be solitary, multiple, or as a form of recurrent respiratory papillomatosis (RRP). Respiratory papillomatosis is mostly seen in the larynx but it can affect any part of tracheobronchial tree. It is rarely isolated in trachea. Distal trachea involvement was reported only in the 5% of RRP cases. In a study, TP is reported in only 5 cases of 15.000 bronchoscopies [1–3]. In our patient, the papillomatous lesion was attached to the tracheal wall with a peduncle and there were no other tracheal or endobronchial lesions.

Although RRP can be seen in all ethnic and age groups, the majority of the reported cases are Caucasian and its distribution is bimodal as in childhood and adulthood. The incidence of RRP in childhood is 43/1.000.000, whereas in adults it is 18/1.000.000. Even though it is thought to be related to vertical transmission at vaginal delivery, there are also a small number of cases reported after cesarean section. Etiology of adulthood RRP is unknown, and it is not certain if the disease is due to sexual transmission or the activation of latent infection [4, 5]. Our patient was 40 years old. He had no prior history of RRP in his childhood or HPV history of his mother.

Tracheal papilloma has no specific clinical presentations and has a wide range of symptoms from cough, shortness of breath at rest or with effort to stridor, and upper airway obstruction. Presentation as bronchial hypersensitivity is also reported in the literature. There is no specific finding at physical examination either [6, 7]. Gao and Cao [8] reported a case with TP similar to our case. Tracheal papilloma occupied 90% of tracheal lumen and physical examination showed that there was a third-degree respiratory distress in their case. In our case, the patient had progressive dyspnea.

The role of imaging in differential diagnosis is limited. Tracheal lesions can be seen at chest X-ray, especially at lateral projection. Chest computed tomography (CT) may identify lesions at trachea and large airways. Fixed or reversible obstruction in the respiratory function tests may aid in diagnosis. The definitive diagnosis is made by FOB and in most cases no other method is needed for diagnosis [9–11]. In our case, tracheal lesion has not been seen on chest X-ray. He was diagnosed by FOB after the lesion was detected at chest CT.

Although TP is known to be benign, there is a very low risk for malign transformation. Malign transformation is more common in HPV-11 than HPV-16 types and its frequency is 0.3–5% [12]. In the follow-up of 244 patients, Naka et al. [2] found the malignity risk as 1.6%. In a study by Barzó et al. [3], carcinoma in situ was detected in 9.4% of 32 patients with laryngeal and tracheal papillomatosis. Xiao et al. [13] reported a case with malign transformation of TP. Reported risk factors that could be related to malign transformation are the radiation exposure, smoking, and bleomycin treatment [14].

Tracheal papilloma treatment is hard and generally requires recurrent endoscopic interventions as well as medical treatment. There are no therapeutic guidelines or randomized study comparing treatment options at this point. Treatment modalities change according to the type, severity, number, and location of the papillomas [15].

In the management of TP, the first aim is to remove the tumor and sustain the airway patency. Since malign degeneration can occur in papillomas, their surgical excision is essential. In RRP, if the lesion is small, segmentectomy can be performed in order to preserve respiratory functions [16]. Various endoscopic surgical methods are successfully used in the management of papillomas. Among these, excision by carbon dioxide laser is most commonly used especially in children [2, 17]. Cryotherapy is also reported to be successful in cases with endobronchial obstruction like our case [18]. If the lesions are limited to a small part of the bronchi, photodynamic therapy, yttrium aluminum garnet (YAG), laser vaporisation, and electrocautery (snare) can be adequate in treatment [19]. In the case of solitary endobronchial papilloma reported by Yıldız et al. [20], a small and localised lesion was resected totally with FOB and forceps biopsies. In two cases of endobronchial papilloma reported by Cömert et al. [21], the lesions were resected by electrocautery snare. Komatsu and Takahashi [22] reported a previously treated TP case that was presented with recurrence after five years and was retreated by KTP/Nd-NAG laser. In our case, since the lesion was large and it caused the obstruction of 80% of tracheal lumen, firstly lumen patency was maintained by forceps biopsies with rigid bronchoscopy and excision of residual tissue with cryotherapy followed.

Local recurrence is common in papillomas. Surgical or bronchoscopic excisions of the lesions are usually not adequate in controlling the disease. For this reason, close clinical follow-up of these patients is very important in the postexcision period. Long and Sani [23] reported that adjuvant therapies (intralesional injection of cidofovir, photodynamic therapy, pulsed dye lase, and indole-3-carbinol) were needed in 10% of RRP cases. Current indications for adjuvant therapy are the requirement of more than four surgical interventions per year, the dissemination of the disease, and the rapid growth of the lesion causing airway compression [24, 25]. In our patient, because the lesion was large and occupied 80% of the tracheal lumen and there was an obstruction risk of the airway in case of recurrence, interferon-*α* (IFN-*α*) treatment was started as adjuvant therapy.

Interferon-*α* (IFN-*α*) is a human leucocyte protein produced naturally as a response to viral infections in the body. It is one of the oldest agents used in the treatment of RRP and its mechanism of action is not fully understood. It binds to receptors at the cells and regulates the cellular metabolism, generating antiproliferative and immune-protective effects. In a multicenter study, solely surgical excision and surgical excision together with IFN-*α* treatment were compared. At the end of the study, regression of the lesions slowed down at the first six months but this effect did not last two years [26]. In a study by Leventhal et al. [27], patients who were treated with IFN-*α* were reexamined 6 months after the initiation of the treatment. In their study, there was 75% complete response in one-third of the patients and it was declared that IFN-*α* treatment can be applied for six months for patients who need surgical excision every two or three months. In a prospective study, patients were followed up for 20 years, it was stated that IFN-*α* treatment was more effective in HPV-6 infection, and there was no adequate response in patients with HPV-11. Also since malign transformation is more common and mortality is higher in patients with HPV-11, determination of type of HPV is advised after the first sampling [28]. In light of these studies, IFN-*α* treatment was applied for six months in our case.

In conclusion, TP is a relatively rare disease that should be closely followed up because of its recurrence and malignant transformation. The present case was successfully treated with cryotherapy and IFN-*α* treatment and his close clinical and bronchoscopical follow-up will continue.
